# Genomics: HapMap Complete

**Published:** 2005-10

**Authors:** Charles W. Schmidt

The International HapMap Project, a consortium of researchers and funding agencies from the United States, Japan, China, Nigeria, Canada, and the United Kingdom, is set to release a dramatically enhanced version of its haplotype map. The newly revised HapMap will be formally introduced on 26 October 2005 at the annual meeting of the American Society of Human Genetics in Salt Lake City. This information will provide researchers with an effective shortcut to map the genes contributing to particular diseases and drug responses.

The HapMap currently characterizes a total of 4 million common DNA sequence variants known as single-nucleotide polymorphisms (SNPs). With the HapMap, scientists are better able to investigate the genetic components of many complex disorders, such as asthma, cancer, and obesity. Mark Daly, an associate member of the Broad Institute, a research collaboration of universities, research centers, and hospitals in Cambridge, Massachusetts, says the HapMap shows where common SNPs are located on human DNA, and how they are distributed among populations in different parts of the world. “The HapMap allows us to accelerate our understanding of genetic variation and its relationship to disease,” he says.

Most SNPs are inherited in blocks, or haplotypes, on the chromosome. Each haplotype typically carries “tag” SNPs that characterize the haplotype as a whole and thus can be used to predict the identity of the other SNPs in the same block. For example, if researchers found that a certain tag SNP showed up consistently in studies of bipolar disorder, that tag could provide some indication of the other nearby SNPs on the chromosome—SNPs that may act in concert to exert some effect on the individual phenotype. Researchers can then look more closely at those neighboring SNPs to see whether and how they contribute to a given disease. The HapMap project has identified 250,000–400,000 such tag SNPs.

Phase I of the project, which was completed in March 2005, characterized 1 million SNPs in the genomes of 269 individuals from four sampled populations: the Yoruba people of Nigeria, Han Chinese from Beijing, Japanese people from Tokyo, and a group in Utah with ancestry from Western and Northern Europe.

In Phase II, the HapMap increased the SNP density characterization in these populations to 4 million. According to Daly, this expanded number encompasses the vast majority of common SNPs thought to exist in human beings.

Lisa Brooks, program director of the Genetic Variation Program at the National Human Genome Research Institute, says that researchers will seek to validate the current HapMap’s findings in additional populations, including African Americans, Mexican Americans, and others. “Phase II has given us a better genomewide HapMap,” she explains. “This is a wonderful resource for mapping genes affecting complex diseases.”

